# Insights into the Voltage Regulation Mechanism of the Pore-Forming Toxin Lysenin

**DOI:** 10.3390/toxins10080334

**Published:** 2018-08-17

**Authors:** Sheenah Lynn Bryant, Tyler Clark, Christopher Alex Thomas, Kaitlyn Summer Ware, Andrew Bogard, Colleen Calzacorta, Daniel Prather, Daniel Fologea

**Affiliations:** 1 Department of Physics, Boise State University, Boise, ID 83725, USA; sheenahbryant@boisestate.edu (S.L.B.); tylerpatrickclark@gmail.com (T.C.); christopherthomas908@boisestate.edu (C.A.T); kware@u.boisestate.edu (K.S.W.); andybogard@boisestate.edu (A.B.); colleenpoulton@u.boisestate.edu (C.C.); danielprather@u.boisestate.edu (D.P.); 2 Biomolecular Sciences Graduate Program, Boise State University, Boise, ID 83725, USA

**Keywords:** lysenin, pore forming toxins, voltage gating, hysteresis, electrostatic screening

## Abstract

Lysenin, a pore forming toxin (PFT) extracted from *Eisenia fetida*, inserts voltage-regulated channels into artificial lipid membranes containing sphingomyelin. The voltage-induced gating leads to a strong static hysteresis in conductance, which endows lysenin with molecular memory capabilities. To explain this history-dependent behavior, we hypothesized a gating mechanism that implies the movement of a voltage domain sensor from an aqueous environment into the hydrophobic core of the membrane under the influence of an external electric field. In this work, we employed electrophysiology approaches to investigate the effects of ionic screening elicited by metal cations on the voltage-induced gating and hysteresis in conductance of lysenin channels exposed to oscillatory voltage stimuli. Our experimental data show that screening of the voltage sensor domain strongly affects the voltage regulation only during inactivation (channel closing). In contrast, channel reactivation (reopening) presents a more stable, almost invariant voltage dependency. Additionally, in the presence of anionic Adenosine 5′-triphosphate (ATP), which binds at a different site in the channel’s structure and occludes the conducting pathway, both inactivation and reactivation pathways are significantly affected. Therefore, the movement of the voltage domain sensor into a physically different environment that precludes electrostatically bound ions may be an integral part of the gating mechanism.

## 1. Introduction

Lysenin, a pore forming toxin (PFT) found in the coelomic fluid of the red earthworm *E. fetida*, induces cytolysis and hemolysis of cells that contain sphingomyelin in their plasmalemma [1,2,3,4,5]. Electrophysiology [2,6,7] and atomic force microscopy [8,9,10] investigations of lysenin inserted into artificial membrane systems have shown that this lytic activity stems from self-insertion of large conducting pores in the target membrane. The physiological role of lysenin is still obscure; nonetheless, lysenin channels possess a great variety of intricate biophysical properties which are commonly shared with ion channels, including large transport rate and selectivity [2,7]. Additionally, lysenin is endowed with unique regulatory mechanisms that set it apart from other PFTs. For example, when reconstituted in artificial membrane systems, lysenin channels show reversible ligand-gating induced by multivalent cations [11,12]. Remarkably, the ligand-induced gating is influenced by the charge density of the ligands; small and highly charged ions (e.g., trivalent metal cations) bind the channel protein at a specific site and induce conformational changes that switch the channel’s conductance between open (fully conducting) and closed (non-conducting), while divalent or voluminous polycations force the channel into a sub-conducting state [11,12]. The macroscopic conductance of the channels is also reversibly modulated by purines (e.g., adenosine phosphates), for which the mechanism of conductance reduction stems from anions binding to a specific site inside the channel’s lumen and impeding the ionic flow [13]. This mechanism of channel occlusion is fundamentally different from ligand-induced gating, which employs a cation-binding site to induce conformational changes.

The most striking feature of lysenin channels is their unique voltage regulation, which has been extensively explored in multiple studies [2,6,7,14,15]. Voltage regulation is a fundamental feature of many PFTs, which generally present symmetrical voltage gating at large transmembrane potentials [16]. In contrast, lysenin channels displays asymmetrical voltage induced gating, which occurs at low transmembrane voltages [6,7] and resembles a basic feature of voltage-gated ion channels. Specifically, lysenin channels are in a high-conductance state (open) for a large range of negative voltages and at low positive voltages. At transmembrane potentials exceeding ~10–20 mV, the lysenin channels transition to a closed conformation characterized by negligible conductance [6,17].

For a population of lysenin channels, the open-close transition is described by a Boltzmann distribution within a two-state model for which the transition is relatively slow, being characterized by a relaxation time of several seconds [17]. The slow response to applied voltages creates premises for dynamic hysteresis in conductance which occur when applying variable voltages that change too fast to be followed by conformational changes of the channels [18]. This non-equilibrium leads to distinct pathways for channel inactivation (channel closing induced by increasing, ascending voltage ramps) and reactivation (channel reopening during decreasing, descending voltage ramps). Dynamic conductance hysteresis, which may be a source of molecular memory, is a fundamental feature of voltage-gated ion channels exposed to oscillatory voltages for which the period of the stimulus is comparable to the relaxation time [18]. However, this phenomenon fails to account for the behavior of lysenin. Lysenin presents a large, static hysteresis in conductance [14,17], which is not common among ion channels or pore forming proteins. While dynamic hysteresis vanishes when the period of the voltage stimulus greatly exceeds the characteristic relaxation time (i.e., when the channels are at equilibrium at any given time during voltage stimulation), lysenin channels retain conductance hysteresis over voltage ramps lasting several hours [17], much larger than their relaxation time.

This unusual feature may be related to the mechanism by which lysenin channels respond to voltage stimuli. In experiments investigating the effects of temperature on lysenin gating [17], it has been found that higher temperatures elicit a strong shift of the voltage-induced gating during ascending voltage ramps. In contrast, temperature has negligible influence on channel reactivation (descending voltage ramps) and the open probability (P_open_) is invariant. This stable reactivation pathway leads to a static hysteresis [17], which could be better understood by gaining more insights into the gating mechanism. This may be possible by considering recent structural data of the lysenin channels [19,20,21], which in conjunction with novel explorations may shed more light on lysenin’s intricate voltage-induced gating and molecular memory.

It has been suggested that lysenin channels alter their conformation by the movement of a gate coupled to a charged voltage domain sensor when under the influence of an external electric field [6], which is functionally similar to many voltage-gated ion channels [22,23,24,25,26,27,28]. Structural data reveals the presence of hinge-like, flexible structures essential for pore formation [19,20,21] which may allow the elusive voltage domain sensor to move. Since both ionic strength and pH strongly modulate the voltage-induced gating of lysenin channels [6], it is natural to assume that the charged voltage domain sensor is exposed to the bulk ionic solution at rest (the state in which all the channels are open at zero transmembrane potential), and screened. To explain the invariant reactivation pathway, we hypothesized that conformational changes, leading to channel closing, move the voltage domain sensor into an environment for which novel physical properties influence how the channels will further respond to voltage stimuli, such as the hydrophobic core of the membrane. Irrespective of different nomenclatures used to define the protein domains, available X-ray and Cryo-EM structural data [19,21] show a mushroom-shaped channel comprising a head and a β-barrel long pore (stem). One may surmise that the voltage domain sensor could be located in the head region since those domains are more prone to movement. In addition, the same structural data indicates the presence of multiple charged sites capable of binding both anions and cations, and lysenin presents such capabilities [11,12,13,29].

The present work was undertaken to produce evidence for the hypothesis that the voltage domain sensor moves into a physically different environment during gating. Our investigations considered electrostatic screening elicited by monovalent and multivalent metal cations acting as counterions for the voltage domain sensor. A simple two-state gating model that assumes a Boltzmann distribution of the states [6,30,31,32] allowed us to estimate the midway voltage of activation (V_0.5_, the voltage at which the P_open_ equals 0.5) and the number n of elementary gating charges in various experimental conditions comprising electrostatic screening induced by counterions. Our results show that electrostatic screening has a major influence on channel inactivation, while the reactivation pathway is basically invariant. However, investigations conducted by employing ATP, which modulate the macroscopic conductance by binding to the channel lumen and partially occluding the conducting pathway [13], show a quantitatively and qualitatively different influence on voltage-induced gating, in support of the hypothesized gating mechanism.

## 2. Results and Discussions

### 2.1. Monovalent Metal Cations Modulate the Voltage-Induced Gating of Lysenin Channels During Inactivation, while Minimally Influencing the Reactivation Pathway

**Figure 1:**
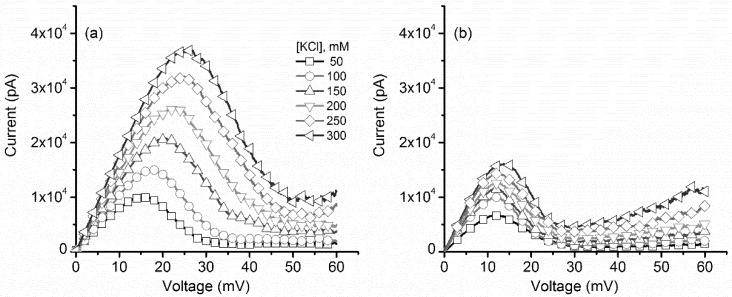
Effects of KCl addition on the I–V characteristics of a population of lysenin channels. (**a**) The I–V plots recorded during ascending voltage ramps indicated changes of the macroscopic conductance (I/V) and voltage required to initiate gating. (**b**) The I–V plots corresponding to descending voltage ramps showed similar changes in the macroscopic conductance of open channels but less dependence of the close-open transition on the ionic concentration, along with hysteresis in macroscopic conductance. Each trace in the panels represents a single, typical run for each particular concentration. All the points in the plots are experimental points; the symbols have been added as a visual aid to discriminate between ionic concentrations.

The macroscopic ionic currents through a large population of lysenin channels, which were inserted into the bilayer membrane and exposed to increasing monovalent ion concentrations, underwent visible changes with regards to their magnitude and voltage required to initiate the open-close transitions during ascending voltage ramps (channel closing, or inactivation, Figure 1a), as our group reported in a previous study [6]. Addition of KCl yielded larger slopes of the linear portion of the current–voltage (I–V) curves recorded at low voltages (i.e., a larger macroscopic conductance, equal to I/V), which follows the increase of the support electrolyte’s ionic conductivity. In addition, we observed a significant shift in the voltage required to initiate voltage-induced gating, which was also dependent on the ionic concentration of the bulk [6]. This shift suggests that the voltage domain sensor of the channel was exposed to the external ionic solution and addition of counterions enhanced electrostatic screening and reduced the gating charge. Consequently, larger transmembrane voltages and electric fields were required to actuate the gate and close the channel. In contrast, the ionic currents recorded for descending voltage ramps (channel reopening, or reactivation, after voltage-induced closing) show both a different qualitative and quantitative response to monovalent ion addition (Figure 1b). To facilitate direct comparison with the ionic currents recorded during ascending ramps, an identical range for the y axis was used to plot the I–V characteristic corresponding to descending voltage ramps. For identical ionic concentrations, the maximal currents recorded during the descending ramps are clearly smaller than what was recorded for the ascending ramps, demonstrating the previously observed hysteresis in conductance [14,17]. In brief, for the same transmembrane voltage, the macroscopic current may have different values depending on the channel’s history (previously open, or previously closed, respectively). Nonetheless, when the decreasing transmembrane voltages approached ~10 mV, the channels fully reopened and their initial conductance was fully reinstated; as inferred from the I–V plots recorded at low transmembrane voltages for all ionic concentrations and ramp directions, the currents and the slopes of the I–V plots at low transmembrane potentials were identical for the two cases, indicating full channel reopening. However, unlike what was observed for ascending voltage ramps, monovalent cation addition did not significantly alter the voltage at which the close-open transition occurred during descending ramps, irrespective of the ionic concentration.

**Figure 2:**
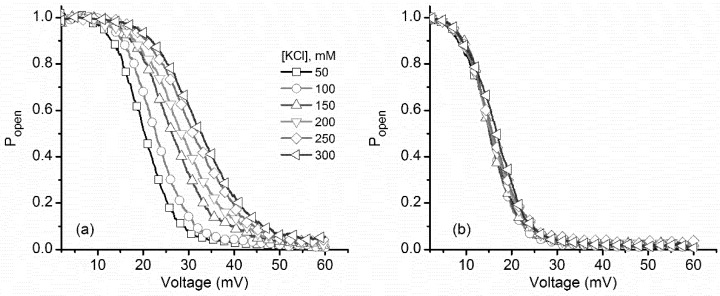
KCl influence on lysenin channels’ experimental open probability. (**a**) The open probability of lysenin channels as a function of voltage underwent a substantial rightward shift for the ascending voltage ramps as the KCl concentration increased. (**b**) In contrast, negligible changes of the open probability occurred during channel reactivation (descending voltage ramps), irrespective of the bulk KCl concentration. Each plot represents a typical curve of the experimental open probability calculated for each particular concentration. All the points in the plots are experimental points, with the symbols added to allow identification of the ionic conditions.

These differences in response to applied voltages, ramp direction, and ionic conditions were also observed in the experimental P_open_ plots (Figure 2), constructed as described in the Materials and Methods section. The very low-voltage experimental points presented large deviations of the P_open_ (due to the very low currents and large noise, which may imply division by near zero numbers), and these points were not represented in the plots. KCl addition yielded a significant rightward shift of the P_open_ for ascending voltage ramps (Figure 2a), while only minor changes were observed for the descending voltage ramps (Figure 2b). These results show a steady, almost invariant channel reactivation pathway, resembling what our group has reported in experiments exploring the effects of temperature on lysenin channel voltage-induced gating [17].

**Figure 3:**
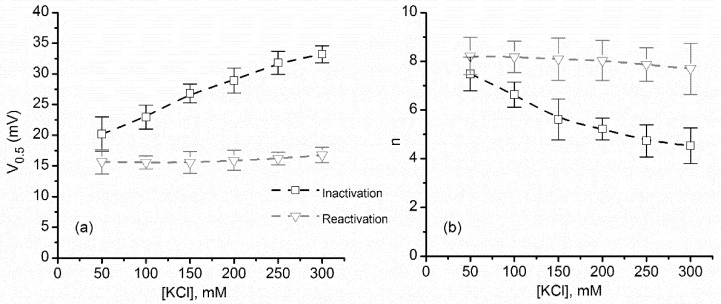
Variation of the midway voltage of activation V_0.5_ and number of elementary gating charges n as a function of KCl concentration. (**a**) The experimental values of V_0.5_ calculated for ascending voltage ramps presented a significant increase with added KCl, while only a weak influence was encountered for descending voltage ramps. (**b**) The values of n calculated from the fit of the Boltzmann distribution equation, for each KCl concentration, suggested strong electrostatic screening of the voltage domain sensor for channel inactivation; only minor changes were estimated for channel reactivation. Each experimental point is represented as mean ± SD from three independent experiments.

For both ramps, we estimated the midway voltage of activation, V_0.5_, from the P_open_ plots [31,33]. Figure 3a shows that V_0.5_ increased monotonically from ~20 mV to ~35 mV upon KCl addition during ascending voltage ramps. As we already surmised from the P_open_ plots, only negligible changes of V_0.5_ were observed for the descending voltage ramps. The V_0.5_ values were next introduced into the Boltzmann distribution equation (see Materials and Methods: Equation (2)) to calculate the number of elementary gating charges n (depicted in Figure 3b).

As predicted from the channel inactivation P_open_ plots, monovalent ion addition led to major variation of the gating charge, which decreased with increasing ionic strength from ~7.5 e to ~4.5 e; the non-linear decrease resembles an exponential decay, in accordance with surface screening [34]. However, the descending voltage ramps yielded a much smaller variation of the gating charge, and were slightly greater (up to ~8 e) for all experimental ionic strengths.

These intricate results show that the history of channel conformation (i.e., closed or open state) influences its further response to applied voltages, which is the essence of molecular memory [17]. This may be realized by dynamic changes in the energy landscape elicited upon conformational changes. The more stable, almost invariant reactivation pathway may provide some clues for understanding the mechanism of the gating process. The strong influence of ionic strength on the gating charge estimated for the inactivation pathway, together with the minimal influence observed during reactivation, suggests that the voltage domain sensor may be exposed to very different environmental conditions during transitions. For a channel in the open state, the voltage domain sensor appears to be exposed to the external electrolyte solution. Therefore, KCl addition promoted electrostatic screening of the gating charge, which led to the rightward shift of the P_open_ during the ascending voltage ramps in a concentration-dependent manner. Channel closure is accompanied by conformational changes and movement of the voltage domain sensor that acts on the gate. This process may result in positioning of the voltage domain sensor into a low polarity environment (i.e., the hydrophobic core of the membrane), from which both water and bound counterions are excluded by the large Born energy penalty [35,36,37,38]. Therefore, irrespective of the ionic strength, the voltage domain sensor will be mostly stripped of electrostatically bound counterions upon gating. Consequently, after channel closing, the voltage domain sensor is characterized by a larger gating charge less influenced by the ionic strength of the bulk solution, which may explain the more stable reopening (reactivation) pathway. This proposed mechanism is well-established for ion channels, for which the movement of a charged voltage domain sensor into the hydrophobic core of the bilayer has been reported [39,40,41,42,43]. Therefore, the “paddle in oil” concept, which consists of large movements of the voltage domain sensor into the hydrophobic core of the membrane [41], is accepted as a valid model of ion channel gating.

### 2.2. Multivalent Metal Cations Influence the Voltage Regulation of Lysenin Channels Similarly to Monovalent Ions

**Figure 4:**
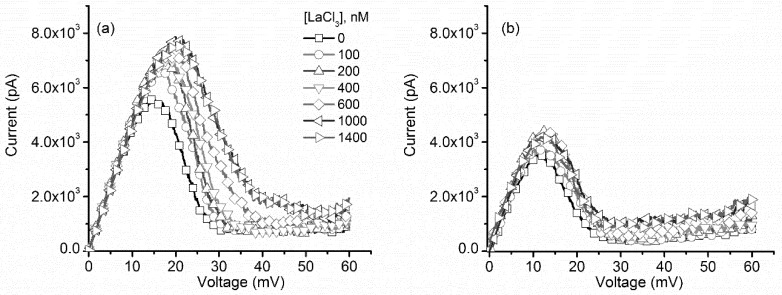
LaCl_3_ influence on the I–V characteristics of lysenin channels. (**a**) The I–V plots recorded for ascending voltage ramps indicated LaCl_3_ induced changes of the voltage required to initiate gating. (**b**) The I–V plots corresponding to descending voltage ramps indicated a minimal influence from the multivalent cations. Each trace in the plots for both panels represents experimental points, with the symbols added as a visual aid.

Electrostatic screening is dependent on ionic strength, which increases with the second power of the electrovalence [44,45], and thus one may expect an even greater influence on the channel’s response upon exposure to multivalent ions. However, our group has reported that multivalent ions induce channel closure by a ligand-induced gating mechanism [11,12], which may introduce roadblocks for such investigations. For example, trivalent metal cations (especially lanthanides) reversibly close lysenin channels and annihilate their macroscopic conductance at ~100 µM bulk concentration [11,12]. Although µmolar addition of La^3+^ may elicit only minor changes of the macroscopic conductance, such small concentrations may not sufficiently screen a gating charge which is simultaneously exposed to substantially larger concentrations of monovalent ions in the support electrolyte. However, it appears that the binding of multivalent ions is more specific and much stronger than monovalent ions [11,12]. This may aid in achieving significant changes of the gating charge without major changes of the macroscopic conductance (owing to significant ligand-induced gating), even upon simultaneous exposure to relatively high concentrations of monovalent ions. Therefore, we investigated the effects of La^3+^ ions on the voltage gating of lysenin channels while using the same experimental approaches adopted for KCl. Addition of small amounts of La^3+^ (no more than 2 µL of various stock LaCl_3_ solutions with concentration in the range 0.1 mM–1 mM for each addition) to the bulk electrolyte (1 mL of 100 mM KCl) to achieve target La^3+^ concentrations (up to 1.4 µM) elicited great changes in the I–V characteristics plotted for ascending voltage ramps (Figure 4a). As we observed that, for the case of KCl, La^3+^ addition required greater transmembrane voltages to initiate voltage-induced gating. For all the La^3+^ concentrations used in these experiments, almost identical slopes of the I–V curves at low transmembrane potentials (the linear, ohmic portion of the curves) indicated that La^3+^ addition elicited negligible changes with respect to the solution conductivity or ligand-induced gating. Comparative analysis of the I–V curves (Figure 4) revealed that, similar to KCl, the voltage required for reopening the channels during descending voltage ramps was smaller than what was required for closing, and that the reopening occurred at similar voltages irrespective of the amount of added La^3+^.

**Figure 5:**
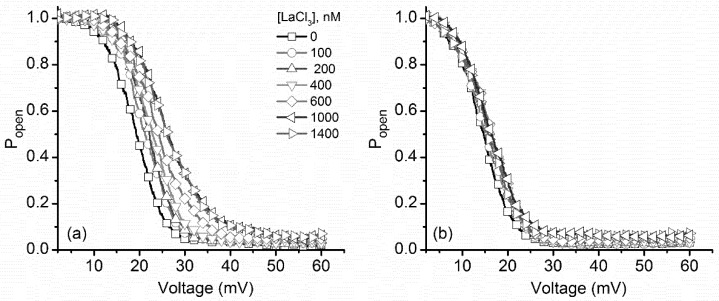
Changes of the experimental open probability (P_open_) of lysenin channels induced by addition of LaCl_3_. (**a**) The voltage-dependent open probability of the lysenin channels shifted substantially rightward for the ascending voltage ramps as the LaCl_3_ concentration increased. (**b**) Conversely, and as with addition of KCl, minor changes were observed during descending voltage ramps. Each trace was constructed from experimental points, with the symbols added for better discrimination between ionic conditions.

These features were also observed in the experimental P_open_ plots constructed for both ascending and descending voltage ramps recorded upon La^3+^ addition (Figure 5). The multivalent metal cations induced a significant rightward shift of the P_open_ for ascending voltage ramps, while the effects presented by the same ions on the P_open_ corresponding to descending voltage ramps were minimal. Therefore, we concluded that the reactivation pathway suffered little influence from the multivalent ions and maintained invariance. In addition, these experimental data show that the influence of La^3+^ manifests even in the presence of much larger concentrations of monovalent ions in the bulk solution, suggesting a greater affinity for their binding sites. We concluded that both monovalent and multivalent cations may compete for the same binding sites since counterion binding is greatly diminished in the presence of very large concentrations of monovalent cations [13].

**Figure 6:**
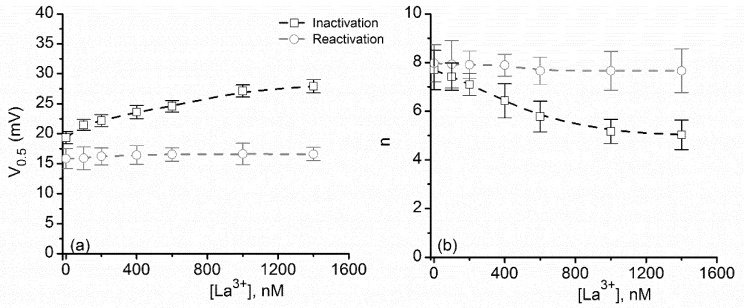
The dependence of V_0.5_ and n on the bulk LaCl_3_ concentration. (**a**) Addition of LaCl_3_ induced significant increases of the V_0.5_ calculated for ascending voltage ramps, while having yielded little change for the descending voltage ramps. (**b**) The total number of elementary charges indicated effective electrostatic screening of the voltage domain sensor upon addition of LaCl_3_ during channel inactivation, while insignificant changes occurred during reactivation. Each experimental point is represented as mean ± SD from three independent experiments.

The experimental V_0.5_ (Figure 6a) and n (Figure 6b) estimated from the best fit of the P_open_ curves for ascending voltage ramps increased significantly (from ~18 mV to ~26 mV) upon sub-µmolar La^3+^ addition, showing great screening effectiveness compared to KCl. Such a result was expected, based on the assumption that both monovalent and multivalent cations elicit ionic screening. Nonetheless, steadier values of V_0.5_ and n were obtained during descending voltage ramps, similar to KCl. This constitutes supplementary evidence for a gating mechanism that implies the movement of the voltage domain sensor into a more hydrophobic environment and supports the hypothesis that multivalent and monovalent cations act similarly, but with different affinities with respect to electrostatic binding to the voltage domain sensor.

### 2.3. ATP Binding to Lysenin Channels Modulates the Voltage-Induced Gating and Affects Both the Inactivation and Reactivation Pathways

The above experiments comprised addition of cations capable of electrostatic interactions with the voltage domain sensor, hence eliciting ionic screening of the gating charge. However, lysenin channels are also capable of interacting with large anions, such as adenosine phosphates, whose interaction manifests as changes in the macroscopic conductance in a concentration dependent manner [13]. Unlike multivalent cations, which most probably bind to a negatively charged site on the voltage domain sensor, purines most likely bind a positively-charged region in channel lumen and elicit physical occlusion of the conducting pathway. Moreover, the binding of purines is a cooperative process, and is most prominent for the highly charged ATP [13]. Therefore, we questioned whether ATP binding to a different site may have a similar influence on the voltage gating profile of lysenin channels, which would challenge the movement of the voltage domain sensor into a more hydrophobic environment as a valid hypothesis.

**Figure 7:**
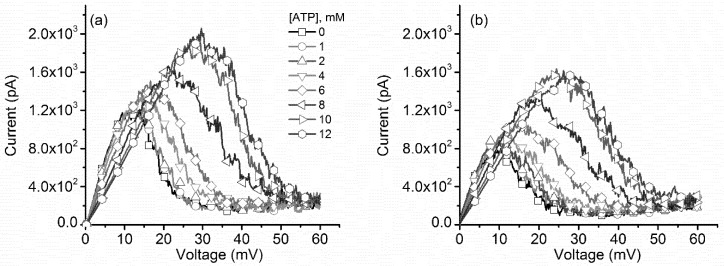
ATP affects the I–V characteristics of lysenin channels in a concentration dependent manner. (**a**) The I–V plots recorded during ascending voltage ramps showed that ATP addition modulated the macroscopic conductance and voltage-induced gating of lysenin channels. (**b**) Opposite to metal cations, ATP induced similar significant changes during descending voltage ramps. Each trace in the panels of the plots represents experimental points, and the symbols have been added to allow easy identification of experimental concentrations.

To answer these questions, we investigated the effects of ATP addition on the lysenin channel voltage-induced gating in similar experiments comprised of ascending and descending voltage ramps. The experimental I–V plots (Figure 7) show that ATP addition to the 135 mM KCl bulk slightly decreased the macroscopic conductance of the open channels in a concentration dependent manner [13], as indicated by the reduced slopes of the linear portions of the I–V curves at low voltages. ATP addition induced a significant shift of the voltage required to close the channels during ascending voltage ramps, similar to monovalent and multivalent metal cations. The response to voltage in the presence of ATP recorded for descending voltage ramps (Figure 7b) was fundamentally different. The hysteresis in conductance remained, and manifested as a different, history-dependent macroscopic current recorded at identical transmembrane voltages. However, the reactivation pathway in the presence of ATP was not invariant, as concluded from the changes observed in the I–V plots.

**Figure 8:**
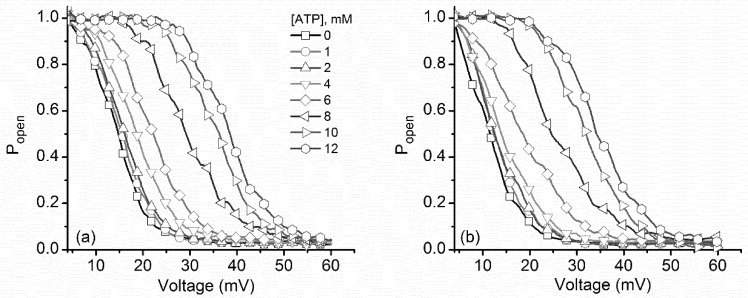
Increased ATP concentration shifted the voltage-dependent open probability of lysenin channels. Addition of ATP induced a rightward shift of the experimental open probability during both (**a**) ascending and (**b**) descending voltage ramps. Each plot represents a typical curve of the experimental open probability calculated for each particular concentration. All the points in the plots are experimental points, and the symbols have been added to allow easy identification of the ATP concentrations. For representation, the P_open_ curves have been smoothed with the Savitsky–Golay protocol (23 smoothing points).

These features are better observed in the experimental P_open_ curves (Figure 8), which were devoid of any influence from the changes in the macroscopic conductance of open channels. Channel inactivation during ascending voltage ramps show a strong rightward shift with ATP addition (Figure 8a), similar to what was observed for both monovalent and multivalent cations. What is striking is that the P_open_ for descending voltage ramps presented a significant concentration-dependent rightward shift (Figure 8b), which is very different from the invariant curves recorded for metal cation addition. Moreover, both I–V and P_open_ plots suggested that a maximal influence of ATP manifests in the 4 mM–10 mM concentration range, while outside this range the induced changes were smaller. This is consistent with the cooperative binding of ATP to lysenin channels, as described in a previous report [13].

**Figure 9:**
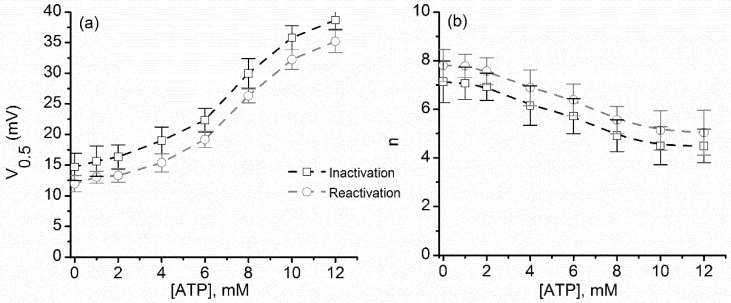
ATP addition alters V_0.5_ and n for both inactivation and reactivation voltage ramps. For the ascending and descending voltage ramps, both V_0.5_ (**a**) and n (**b**) varied with the concentration of ATP. Each experimental point is represented as mean ± SD from three independent experiments.

Differences between metal cations and ATP were also observed in the estimated V_0.5_ and n calculated at different concentrations (Figure 9). Both V_0.5_ and n followed similar patterns of change with ATP concentration, as expected from the summary analysis of the P_open_ and I–V plots. Also, the particular sigmoidal shape of the plots is in agreement with the cooperative binding of ATP to lysenin [13]. Interestingly, the V_0.5_ value measured at 0 mM ATP (i.e., ~12 mV) was the smallest recorded from all the experiments described in this work. This may be explained by considering the consistently lower number of channels inserted in the membrane for the ATP experiments; previous studies reported that crowding in the membrane plane (achievable at large channel densities) may influence the voltage gating of lysenin channels [15]. In the case of the ATP experiments, a different lysenin batch was used, which consistently provided a lower number of inserted channels that gated at lower transmembrane voltages.

We presented evidence in support of the hypothesis that the voltage domain sensor of the lysenin channel moves into a different physical environment upon voltage-induced gating. Based on earlier evidence presented for ion channels, we assumed that the gating charge, exposed to the aqueous environment at rest, may penetrate deep into the membrane and exclude the bound ions. Before closing, the voltage domain sensor is electrostatically screened by cations; therefore, ionic strengths significantly modulate the voltage-induced gating. Channel reopening after closing comprises a voltage domain sensor which is minimally screened and results in a more stable reactivation pathway. From investigating the influence of ATP on voltage gating, we concluded that anions act in a very different manner than cations because they do not bind to the same sites of the channel. The cations bind to the voltage domain sensor, which then undergoes conformational changes and penetrates into a more hydrophobic environment from which water and ions are excluded. For ATP, which binds a different site, the influence on the voltage induced gating may be explained by long range electrostatic interactions between the bound anions and the voltage domain sensor, which may manifest even when the voltage domain sensor is in a nonpolar environment. The proposed gating mechanism is supported by experimental data but we may not overlook some experimental and theoretical limitations. In this work, we consistently used a reasonably-long period for the driving voltage stimulus (20 min), which is much larger than the characteristic relaxation time of lysenin channels (seconds). However, addition of cations or anions may substantially change the relaxation time, and therefore the I–V and P_open_ plots may not represent true steady states. Nonetheless, in such situations, the channels that closed at lower voltages resided for longer times in the closed state, yet the reactivation pathway was invariant. Also, the Boltzmann distribution considered in this work (and similar approaches [31]) did not account for dynamic changes of the energy landscape during gating. In spite of these limitations, both the similarities and differences between the voltage-induced gating of lysenin channels exposed to cations and anions support a model in which the voltage domain sensor moves into the membrane, and may explain the persistent hysteresis in conductance.

## 3. Materials and Methods

### 3.1. Bilayer Lipid Formation, Channel Insertion, and Ionic Addition

The vertical bilayer membrane chamber consisted of two polytetrafluoroethylene (PTFE) reservoirs (~1 mL volume each) separated by a thin PTFE film in which a small hole (~ 70 µm diameter) was pierced by an electric spark. The reservoirs were filled with electrolyte solutions (50 mM KCl, if not otherwise noted, buffered with 20 mM HEPES, pH 7.2) and connected to the Axopatch 200B electrophysiology amplifier (Molecular Devices, San Jose, CA, USA) via Ag/AgCl electrodes. The analog signal of the amplifier was further digitized and recorded with the DigiData 1440A digitizer controlled by the pClamp 10.6.2.2 software package (Molecular Devices, San Jose, CA, USA, 2016). The bilayer was produced using a mixture of 1 mg asolectin (Sigma-Aldrich, St. Louis, MO, USA), 0.4 mg cholesterol (Sigma-Aldrich, St. Louis, MO, USA), and 0.5 mg sphingomyelin (Avanti Polar Lipids Inc., Alabaster, AL, USA) in 100 μL n-decane (TCI, Portland, OR, USA). The bilayer integrity was verified by both capacitance and conductance measurements. Channel insertion was performed by addition of small amounts of lysenin (0.3 pM final concentration, Sigma-Aldrich, St. Louis, MO, USA) to the ground reservoir under continuous stirring with a low noise magnetic stirrer (Warner Instrument, Hamden, CT, USA). Individual channel insertion was monitored by observing the discrete, step-wise changes of the ionic current through the membrane biased by − 60 mV transmembrane potentials [7]. After completion of insertion, observed as a steady value of the ionic current, the free lysenin was removed by flushing the ground reservoir with fresh buffered electrolyte. Lysenin gating at positive potentials was observed from I–V plots recorded in the range 0 mV: + 60 mV by exposing the channel-containing membrane to voltage ramps created with the digitizer. We observed that different batches of lysenin presented slight differences with respect to voltage-induced gating (i.e., variations of the voltage required to initiate gating), therefore we used identical batches of lysenin for each independent experiment focused on investigating the influence of a particular ion on the gating profile.

Stock solutions of KCl (Fisher Scientific, Pittsburgh, PA, USA), LaCl_3_ (Alpha Aesar, Tewksbury, MA, USA), and ATP (Sigma-Aldrich, St. Louis, MO, USA) were prepared by dissolving the powders in 20 mM HEPES (pH 7.2). To achieve the target concentrations in the bulk, corresponding amounts of stock solutions were added to both reservoirs under continuous stirring. This procedure was repeated for all target concentrations achieved for the experiments.

### 3.2. Data Collection, Analysis, and Mathematical Modelling

For each ionic concentration, the voltage-induced gating of lysenin channels was investigated by recording the ionic currents in response to linear, triangle-shaped voltage ramps. The ramps were created with the pClamp 10.6.2.2 software package and delivered to the electrophysiology amplifier via one of the digitizer’s outputs. Each voltage ramp stimulus had a period of 20 min (10 min for ascending, and 10 min for descending) in the range 0 mV: + 60 mV, at a sampling rate of minimum 1 s, with a 1 kHz hardware filter. Each recording was saved as a separate file and further analyzed with Origin 8.5.1 (OriginLab, Northampton, MA, USA, 2011), pClamp 10.6.2.2 and Mathematica 10.4 (Wolfram Research, Champaign, IL, USA, 2016).

For analysis and modelling, we considered that the voltage-induced gating of lysenin channels is described by a two-state model [6]:

(1) Open⇔Close

Within this model, the open probability P_open_ of the channels in response to applied voltages is described by the Boltzmann distribution [31,46]:

(2) $$P_{open}=\frac{1}{1+e^{\frac{-neF(V_{0.5}-V)}{RT}}}$$

where *n* is the total number of elementary gating charges *e* of the voltage domain sensor (e = 1.6 × 10^−19^ C), V_0.5_ is the midway voltage of activation (the voltage at which P_open_ = 0.5), V is the applied voltage, R is the universal gas constant (8.34 J/K), F is Faraday’s number (F = 96485.33 C/mol), and T is the absolute temperature (T = 295 K).

Within the two-state model, the experimental open probability for each applied voltage was derived from [23,32,47]:

(3) $$P_{open}=\frac{I}{I_{\max}}$$

where I is the ionic current measured for a particular voltage, and I_max_ is the ionic current that would be recorded at the same voltage if all the channels were open. For each particular voltage, I_max_ was estimated from a straight line constructed by fitting the linear portion of each I–V curve recorded at low transmembrane voltages (when all the channels are open).

The midway voltage of activation was experimentally determined from the P_open_, plotted for each of the ionic conditions, and further used in the theoretical model to determine n. Since even a closed lysenin channel may present a small leakage current [6,17], this was corrected in the experimental P_open_ only for the two highest KCl concentrations used in our experiment, which presented visible leakages. No other correction was performed for experimental data. All graphs have been prepared using the Origin 8.5.1 software.
